# Effects of Physical Activity and Mindfulness on Resilience and Depression During the First Wave of COVID-19 Pandemic

**DOI:** 10.3389/fpsyg.2021.700742

**Published:** 2021-07-29

**Authors:** Roberta Antonini Philippe, Laurie Schwab, Michele Biasutti

**Affiliations:** ^1^PHASE Lab, Institute of Sport Sciences, University of Lausanne, Lausanne, Switzerland; ^2^Institute of Psychology, University of Lausanne, Lausanne, Switzerland; ^3^Department of Philosophy, Sociology, Education, and Applied Psychology, University of Padova, Padua, Italy

**Keywords:** COVID-19, resilience, depression, physical activity, mindfulness

## Abstract

The first wave of the COVID-19 pandemic generated a significant number of stressors that the Swiss population had to deal with. In order to cope with and adapt to such adversity, it is essential to have protective factors that allow for resilience. The objective of this study was to investigate the effects of mindfulness and physical activity on depression and resilience during the first wave of the COVID-19 pandemic. A quantitative method was adopted asking participants who were engaged in physical activity or mindfulness to fill a battery of measures of depression and resilience and some demographic questions. The results showed that mindfulness practice strengthened the initial level of resilience of practitioners, suggesting that mindfulness meditation is a tool for coping with adversity during a potentially traumatic event. Conversely, physical activity practitioners maintained a stable resilience score over time, suggesting that exposure to adversity did not disrupt their state of biopsychospiritual homeostasis. Moreover, being physically active decreased the depression score over time. Regarding demographic variables, gender differences were observed in the average scores in the resilience scale and in the Depression Inventory.

## Introduction

The beginning of the year 2020 was marked by the appearance and global spread of COVID-19 pandemic. While no pharmacological treatment was available to defeat this new virus, authorities around the world were forced to establish drastic preventive measures that disrupted the daily lives of billions of people. The main precautions included recommendations for hygiene, social distancing, restriction of interpersonal contact, and even quarantine of cities, regions, or entire countries (Antonini Philippe et al., 2020). In Switzerland, the authorities prescribed a semi-lockdown, recommending residents only go on basic outings, such as those for food and vital goods, work or medical consultations. However, in case of absolute necessity, citizens were allowed to take a breath of fresh air while complying with the health regulations, otherwise they could be fined.

The suppression of sports and cultural events, the ban on gatherings and the closure of schools, entertainment, and workplaces brought social life to an almost complete halt (Biasutti et al., 2021; Schiavio et al., 2021). Economic losses, lack of resources for medical care or inaccessibility to necessities can cause many community problems. The urgency of this health crisis can negatively affect the health, safety, and well-being of the population, leading to insecurity, confusion, emotional isolation, or stigmatization. These various identified stressors can result in several negative emotional reactions such as generalized distress or psychological disorders (Pfefferbaum and North, 2020). In this sense, the COVID-19 pandemic can be seen as a disaster, which is defined as “a serious disruption of the functioning of a community or society causing widespread human, material, economic or environmental losses that exceed the capacity of the affected community or society to cope with them using its own resources” (International Strategy for Disaster Reduction, 2004, cited in Makwana, 2019). Disasters are typically measured in terms of social and economic losses and costs, and the emotional suffering experienced by individuals at the heart of the disaster. The negative impact of the disaster on the well-being and mental health of human beings is usually manifested in post-traumatic stress disorder (PTSD). Although a natural disaster such as the COVID-19 viral infection does not meet all the criteria necessary to be diagnosed as PTSD, other psychological disorders may emerge such as anxiety or depression (Makwana, 2019).

In the context of COVID-19 where the Swiss population was in a semi-lockdown, certain individual outdoor physical activities were tolerated, such as walking, running, or cycling, when they respected the health measures in place. Physical activities as well as exercises and sports are known to bring numerous benefits to both physical and mental health. Thus, engaging in such practices on a regular basis would contribute to emotional well-being and reducing symptoms of anxiety and depression while improving the mood of practitioners (Penedo and Dahn, 2005). In this sense, the practice of mindfulness meditation can have beneficial effects on the health of its practitioners (Brown and Ryan, 2003; Grossman et al., 2003). Engaging in physical activity or practicing Mindfulness are supposed to promote, aspects such as the physical, mental and/or emotional well-being of their practitioners.

### Resilience

Resilience can be defined as “the personal qualities that enable others to thrive in the face of adversity” (Connor and Davidson, 2003, p. 76, cited in Fletcher and Sarkar, 2013). During their lives, many people are confronted at least once with a potentially traumatic event (PTE) such as a physical or sexual assault, an accident or a natural disaster (Kessler et al., 1995). However, in many cases, individuals are resilient and develop little or no mental illness (Kessler et al., 1995; Bonanno et al., 2007; Makwana, 2019). Events that can be considered particular or uncommon to normal daily experiences are said to be “potentially traumatic” because they do not affect everyone in the same way (Bonanno and Mancini, 2008). For a long time, it was assumed that a person exposed to a traumatic event would react to it in a binary way: either they would have PTSD or they would not. However, through empirical studies of individual variations in PTSD, different unique and variable trajectories of the consequences of exposure to such events were highlighted. The following four possible trajectories were considered: (1) the individual develops chronic dysfunction, which characterizes the development of chronic psychopathologies that actually affect only a tiny fraction of those exposed; (2) the individual may have delayed reactions and therefore reacts later in a negative way to PTE; (3) because of their resilience, and, therefore, their coping skills in the face of adversity, some people exposed to PTE do not develop any form of psychopathology; and (4) the individual recovers and regains a basic level after having lived for a time with a certain level of pathology (Bonanno and Mancini, 2008). The possibility that a person is resilient in the face of a potentially traumatic event does not mean that he or she cannot experience some stress reactions to the situation experienced. These reactions are usually mild to moderate, do not persist over time and do not significantly interfere with the individual’s ability to function (Bonanno and Mancini, 2008).

According to Connor and Davidson (2003), human beings have a homeostatic biopsychospiritual balance point that allows them to adapt their bodies and minds to the circumstances of the life they are living through. An individual’s ability to cope with the internal and/or external stressors he or she encounters will be influenced by the successful and unsuccessful adaptive experiences he has previously had. Certain experiences can lead to a rupture of biopsychospiritual homeostasis requiring a reintegration process, which can lead to four different results: (1) the disruption gives the individual the opportunity to grow and improve his or her resilience capacities, thus enabling he or she to reach a new and higher state of homeostasis; (2) a return to basic homeostasis level after having invested an effort to overcome the disruption; (3) the individual has difficulty recovering from the disruption, resulting in a lower state of homeostasis than initially; (4) the attainment of a dysfunctional state in which poor strategies, such as self-destructive behaviors, are used in an attempt to cope with the disruption (Connor and Davidson, 2003).

Rutter (1985) defines a protective factor as “an influence that modifies, enhances or alters a person’s response to an environmental hazard that predisposes him or her to an inappropriate outcome” (Rutter, 1985, p. 600). Early studies that looked at factors protecting a person exposed to a PTE concluded that protective factors could be individual (e.g., temperament) or socio-contextual (e.g., social support) (Cowen, 1991; Werner, 1995). Demographic variables may also play a role: it is assumed that older, well-educated Caucasian men have higher levels of resilience, and vice versa (Bonanno et al., 2007). Social and material resources also appear to play a key role in resilience and several researchers have highlighted the usefulness of these types of resources in coping with stress (Lazarus and Folkman, 1984; Holahan and Moos, 1991). In the context of COVID-19, different protective factors have been specifically put forward to promote resilience regardless of the degree of exposure to the virus encountered by individuals across the globe. These factors included: optimism, social connectedness and support, staying informed about the situation while keeping a distance from the information received, distraction and decreasing social isolation through connected communication channels (Chen and Bonanno, 2020).

Resilience appears to be positively correlated with positive mental health indicators and vice versa. In other words, the higher an individual’s level of resilience, the higher is his or her level of life satisfaction and sense of positive affect. Conversely, the lower an individual’s level of resilience, the higher his or her scores for depression, anxiety and various negative affects. The level of resilience can vary according to age, gender and adversity encountered (Hu et al., 2010). Davydov et al. (2010) proposed a model of resilience in the form of a three-mechanism system. Resilience would promote good mental health by protecting it from the negative effects of adversity or reducing its negative impacts. Thus, a high level of resilience would allow individuals to better respond to a situation that could potentially harm their mental health (Davydov et al., 2010).

Resilience is a modifiable construct and can therefore be improved (Southwick et al., 2011). The third wave of research on resilience is focused on the development and evaluation of interventions aimed at promoting or strengthening psychological resilience and preventing stress-related dysfunction. There is no theoretical framework that has been empirically tested and can be relied upon. However, resilience appears to be determined by several modifiable factors: interventions to strengthen resilience should therefore target the strengthening of these factors (Helmreich et al., 2017).

### Depression

The World Health Organization (WHO) defines depression as “a common mental disorder characterized by sadness, loss of interest or pleasure, feelings of guilt or worthlessness, disturbed sleep or appetite, fatigue, and difficulty concentrating” (World Health Organization (WHO), 2017). According to the Diagnostic and statistical manual of mental disorders 5th ed. (DSM-V), it is specified that to be diagnosed as a characterized depressive disorder, a minimum of five of the symptoms listed above must be present and must persist for a minimum of two consecutive weeks (American Psychiatric Association (APA), 2013). This disorder accounts for 10% of the non-fatal diseases affecting the most people in the world. In addition, major depressive disorders affect women more than men, with an estimated global prevalence of 5.8% in women and 3.5% in men in 2013 (Salk et al., 2017). According to the WHO, in 2015, more than 300 million people worldwide suffered from depression, representing the equivalent of 4.4% of the world’s population, regardless of age, gender, and ethnicity (World Health Organization (WHO), 2017). Depression can be diagnosed in primary care and then treated with psychotherapy and pharmacological treatments with an estimated 60–80% efficiency. However, it is estimated that only 10–25% of individuals with depression would receive treatment for their condition. This low rate of treatment is thought to be due to a lack of resources and/or trained professionals, but also because of the social stigma associated with the condition (Dinas et al., 2011).

### Physical Activity

Physical activity is defined as any bodily movement produced by skeletal muscles that require energy expenditure (World Health Organization (WHO), 2018). Regular physical activity is proven to help prevent and manage several diseases and can improve mental health, quality of life, and well-being (Antonini Philippe et al., 2019). Physical activity could be an easily implementable method to build resilience and protect individuals from adversity such as psychological distress or psychological disorders. In fact, physical activity has been highlighted for its effectiveness in preventing and improving certain psychological disorders such as depression (see for example, Conn, 2010; Cooney et al., 2013). Several hypotheses could explain why physical activity would have such kind of benefits. The first hypothesis is biological and is related to neurotransmitters such as serotonin, beta-endorphins, or dopamine released during while being physically active (Ernst et al., 2006; Ho et al., 2015). The second hypothesis relates to social factors; indeed, physical activity could promote connectivity and the creation of social ties if we consider that many kinds of physical activities, physical exercise, or sports are, above all, social activities where it is therefore possible to socialize. Thus, “increasing social capital could provide support against stressors and improve the mental health of individuals” (Ho et al., 2015, p. 2). The third and last hypothesis concerns the psychological dimensions; the challenging aspect of physical activity would help the individual develop self-confidence and certain capacities, such as self-efficacy and resilience. Physical activity would improve general well-being and decrease the risk of developing mental pathologies (Ho et al., 2015). Ho et al. (2015) attempted to empirically test the psychological and social factor hypotheses to establish the link between physical activity and the psychological well-being of children and adolescents. They concluded that it is the psychological dimensions, specifically resilience, that seem to mediate the beneficial link between physical activity and psychological well-being. In fact, resilience accounted for nearly 60% of this relationship and could be explained by the “challenge model” (Ho et al., 2015). This model proposes to consider the link between risk (e.g., stress factors) and negative outcome (e.g., depression) as a curvilinear relationship such that a moderate and manageable risk would lead to a better long-term outcome than no risk or a risk that is too great and unmanageable (Garmezy et al., 1984).

Physical activity has also been shown to be effective in building resilience in several populations exposed to high levels of stressors such as teachers and nurses (Ozkara et al., 2016; Yu et al., 2020). Elite athletes also have often an above-average level of resilience, which may be due to the many challenges to which they are exposed. To cope with adversity, athletes would have the ability to assess stressors in a positive way: they would see them as an opportunity for learning, mastery, and development (Fletcher and Sarkar, 2012).

### Mindfulness

Another practice that has shown beneficial effects on the mental and physical health of its practitioners is mindfulness. Mindfulness could be defined as a form of meditation focuses on human experience and, more specifically, on consciousness (Brown et al., 2007). Thus, the goal of mindfulness meditation is to intentionally focus attention on the “here and now,” on immediate experience without judgment (Kabat-Zinn, 2003). Mindfulness has its origins in Buddhist psychology and shares certain conceptual ideas with several philosophies and psychological traditions such as ancient Greek philosophy, phenomenology, existentialism, and naturalism in later Western European thought, as well as transcendentalism and humanism in America (Brown et al., 2007).

The benefits of mindfulness can be categorized in the following three dimensions: emotional benefits, interpersonal benefits, and intrapersonal benefits.

The first is the emotional benefits, which include emotion regulation, decreased reactivity to emotions, and increased flexibility of response (Davis and Hayes, 2011). The practice of mindfulness would improve an individual’s ability to cope with emotional challenges as well as his or her tolerance of negative affects (Farb et al., 2010). The mechanisms by which these improvements would occur could be related to the fact that Mindfulness promotes metacognition, awareness, and decreased rumination through disengagement from persistent negative cognitive activities. In addition, mindfulness is believed to transform individuals’ attention abilities through working memory gains that participate in effective emotion regulation strategies (Davis and Hayes, 2011). Mindfulness would therefore improve the regulation of emotions experienced by individuals by influencing their awareness of their emotional experiences (Hill and Updegraff, 2012). They would thus react less to negative affects and have more cognitive flexibility (Davis and Hayes, 2011). Cognitive flexibility can be understood as “the human ability to adapt cognitive processing strategies to cope with new and unexpected conditions” (Moore and Malinowski, 2009, p. 177). The second dimension on which Mindfulness has a beneficial effect concerns interpersonal benefits. A high level of mindfulness would have a positive impact on individuals’ satisfaction with romantic relationships and would also allow them to better respond to the stress emanating from their relationships (Barnes et al., 2007). The third and final dimension on which Mindfulness would act concerns intrapersonal benefits. Indeed, Mindfulness seems to reduce perceived stress as well as psychological distress, thus partially improving psychological well-being (Carmody and Baer, 2008).

There are different approaches that assume that increasing one’s consciousness would provide a more truthful perception, reduce negative affects, and improve vitality and adaptation to difficult life situations (Grossman et al., 2003). According to Brown and Ryan (2003), mindfulness can facilitate well-being by adding clarity and vividness to the experience of the moment. It would also allow for closer contact with the present moment by eliminating potentially discriminatory thoughts that may taint the lived experience (Brown and Ryan, 2003).

## Materials and Methods

### Aims and Ethics

The current study aims to investigate the effects of mindfulness and physical activity on depression and resilience during the first wave of the COVID-19 pandemic. The first objective was to examine the evolution of the resilience and depression score according to the type of practice namely physical activity or mindfulness. The second objective was to verify the impact of gender on the depression and resilience scores. The study was conducted in accordance with the Declaration of Helsinki and the Code of Ethics and Conduct of the British Psychological Society. All participants were informed about the anonymization of the data, and about each procedural step of the research, from data collection to analysis and publication. Ethical approval for the recruitment of questionnaire respondents was granted by the Research Ethics Committee of the University of Graz.

### Study Design and Recruitment

**FIGURE 1:**
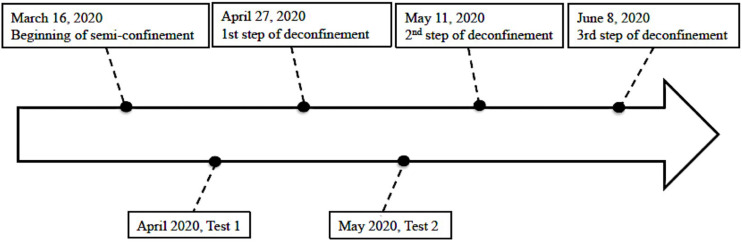
Flow chart of the study.

This paper presents a longitudinal study conducted during the first wave of the COVID-19 pandemic in Switzerland, from mid-March to mid-May 2020 as reported in Figure 1. A quantitative method was adopted asking participants who were engaged in physical activities or mindfulness activities to fill a battery of measures of depression and resilience and some demographic questions in two different moments.

Recruitment of participants was done at the beginning of April 2020, through an e-mail offering people, who were known to be engage in physical activity or mindfulness during semi-lockdown, to voluntarily participate in this study. The e-mail included the link to access the questionnaire on Surveyhero and was accompanied by a short message explaining the purpose of the research, the inclusion criteria (i.e., people over 18 years and physically active or practicing mindfulness) and the guarantee of the confidentiality and anonymity of all data collected. Participants could choose whether they wanted to be contacted to complete the questionnaire a second time, which was done in May 2020, i.e., 4–6 weeks later.

### Data Collection

The data collection was conducted in two phases through an online questionnaire consisting of 45 questions and sought to measure participants’ depression and resilience scores using two tools:

(a) The French version of the Connor and Davidson Resilience Scale (f-CD-RISC) is a self-report questionnaire to measure resilience. This questionnaire is composed of 21 items structurally organized into three factors which are (1) tolerance of negative affects; (2) tenacity; and (3) self-confidence. The items are presented in the form of a five-point Likert scale ranging from 0 (not at all) to 4 (almost all the time). All items are proposed as affirmations (example of item 15: “I prefer to take matters into my own hands to solve problems that arise rather than letting others make all the decisions”) (Guihard et al., 2018).
(b) The Beck Depression Inventory-Fast Screen-France (BDI-FS-Fr) is also a self-report questionnaire composed of seven items (Beck et al., 2000). This questionnaire targets indicators that are not somatic and is very simple to complete since it only takes about 2 min to complete. When completing the questionnaire, the respondent is asked to choose the answer that best corresponds to his or her state over the last two weeks concerning: (1) sadness; (2) pessimism; (3) failures in the past; (4) loss of pleasure; (5) negative feelings toward himself/herself; (6) critical attitude toward himself/herself; and (7) thoughts or desires for suicide. Responses are organized into four points ranging from 0 to 3 for each item (Alsaleh and Lebreuilly, 2017).

The questionnaire also contained demographic and informational questions about the participant and his or her physical activity or mindfulness practice (i.e., type of practice before and during lockdown, reasons for practicing, and duration per day).

### Data Analysis

The data was collected on Surveyhero and then analyzed using IBM SPSS Statistics in its 26 version for Windows (Ibm Corp. Released, 2019). First, a descriptive analysis of the data was carried out in order to get a brief idea of the results obtained. The comparison of the initial level of resilience between the two practice groups and independently of gender was tested using a Student’s parametric *t*-test, while that of depression was tested using a non-parametric Mann–Whitney U-test. Subsequently, the influence of group or gender on the change in depression score over time was analyzed using Wilcoxon’s signed rank tests, while the influence of group or gender on the evolution of the resilience score over time was analyzed using a repeated measures ANOVA with an intra-subject factor and Greenhouse-Geisser correction. In addition, a *post hoc* test of Tukey was conducted to control which group or gender was affected by time. A linear regression was conducted to determine whether the level of resilience could explain some of the variance in the depression score.

## Results

A total of 195 participants took part in the first phase of the study, whose distribution can be read in Table 1.

**TABLE 1:** Distribution of the number of participants by practice and by questionnaire in both phases of the study.

	Group	Gender	BDI T1	BDI T2	RISC T1	RISC T2
N	PA	Female	100	51	100	51
		Male	47	19	47	19
	Mind	Female	38	9	38	9
		Male	10	6	10	6

*PA, Physical Activity; Mind, Mindfulness; BDI T1, Beck’s Depression Inventory at time 1; BDI T2, Beck’s Depression Inventory at time 2; RISC T1, Connor and Davidson Resilience Scale at time 1; RISC T2, Connor and Davidson Resilience Scale at time 2.*

Thus, 147 participants were engaged in physical activity (i.e., 100 women; 47 men) while 48 participants were practicing mindfulness (i.e., 38 women; 10 men) during the first wave of COVID-19 pandemic. In the second phase, 85 participants, including 70 participants who were engaged in physical activity (i.e., 51 women; 19 men) and 15 participants who practiced mindfulness (i.e., 9 women; 6 men) completed the survey again. The age of the participants as well as the main results obtained by the Physical Activity (PA) group and the Mindfulness (Mind) group on the CD-RISC and the BDI-FS-Fr at the first (T1) and second (T2) cut-off times and by gender are summarized in Table 2. Descriptive data are presented as means, medians, and standard deviations.

**TABLE 2:** Descriptive overview of participants’ demographics and results.

	Group	Gender	Age T1	Age T2	BDI T1	BDI T2	RISC T1	RISC T2
Mean	PA	Female	24.0	22.6	2.83	2.55	59.2	58.6
		Male	26.2	37.6	1.26	0.684	63.9	65.9
	Mind	Female	34.6	23.6	2.74	2.67	57.1	54.6
		Male	36.0	39.8	2.40	0.333	54.3	64.0
Median	PA	Female	23.0	22.0	2.00	2.00	58.5	58.0
		Male	25.0	28.0	1.00	1.00	64.0	65.0
	Mind	Female	28.0	25.0	2.00	3.00	58.0	58.0
		Male	31.5	36.0	1.50	0.00	52.5	63.5
Standard deviation	PA	Female	6.20	2.31	3.03	2.48	11.2	10.4
		Male	8.40	15.4	1.62	0.946	7.81	9.04
	Mind	Female	14.3	2.89	2.68	2.83	10.5	10.6
		Male	16.6	19.6	2.84	0.516	10.0	7.54
**Shapiro–Wilk test of normality**								
Statistics	PA	–	–	–	0.811	0.819	0.982	0.973
	Mind	–	–	–	0.869	0.742	0.984	0.927
df	PA	–	–	–	147	147	70	70
	Mind	–	–	–	48	48	15	15
Sig.	PA	–	–	–	0.000	0.000	0.057	0.131
	Mind	–	–	–	0.000	0.001	0.742	0.248

*PA, Physical Activity; Mind, Mindfulness; BDI T1, Beck’s Depression Inventory at time 1; BDI T2, Beck’s Depression Inventory at time 2; RISC T1, Connor and Davidson Resilience Scale at time 1; RISC T2, Connor and Davidson Resilience Scale at time 2.*

The normality distribution of the means of the f-CD-RISC and BDI-FS-Fr scores obtained by the different groups at the two passing times was verified using the Shapiro-Wilk test, the results of which are shown in the Table 2. The data collected in relation to the resilience scale follow the normality law [W(147) = 0.982, *p* = 0.057 for PA at RISC T1; W(48) = 0.984, *p* = 0.742 for mindfulness at RISC T1; W(70) = 0.973, *p* = 0.131 for PA at RISC T2; W(15) = 0.927, *p* = 0.248 for mindfulness at RISC T2], while the data collected on the depression inventory does not follow it [W(147) = 0.811, *p* = 0.000 for PA at BDI T1; W(48) = 0.869, *p* = 0.000 for mindfulness at BDI T1; W(70) = 0.819, *p* = 0.000 for PA at BDI T2; W(15) = 0.742, *p* = 0.001 for mindfulness at BDI T2].

The main results of the study are shown in Table 3. The results of Student’s *t*-test in the first phase [*t*(193) = −2.19, *p* = 0.030] of the assessment indicated that men and women had a significant difference in the average of the f-CD-RISC scores they obtained, regardless of the group they were part of. Women had a significantly lower resilience scores than men. An additional Student’s *t*-test was conducted to test the means of the resilience scores recorded by the two practice groups at the first pass time which showed that mindfulness practitioners had a significantly lower resilience score than activity practitioners [*t*(193) = 2.40, *p* = 0.017]. Homogeneity of variance was always respected [*F*(1,193) = 3.32, *p* = 0.070]; [*F*(1,193) = 0.086, *p* = 0.770].

**TABLE 3:** Overview of the main results of the study.

		Resilience			
		***t***	**df**		***p***
Initial intersex difference		-2.19	193		0.030
Initial intergroup difference		2.40	193		0.017
**Evolution over time**		**F(1,83)**		***p***	
Group		4.322		0.041	
Gender		2.36		0.129	
Time		12.691		< 0.001	

		**Depression**			

		**U**		***p***	
Initial intersex difference		2911		0.004	
Initial intergroup difference		3227		0.364	
**Evolution over time**		**Z**		***p***	
Group	PA	-1.983		0.047	
	Mind	-1.274		0.203	
Gender	Male	-4.006		<0.001	
	Female	-6.591		<0.001	

*PA, Physical Activity; Mind, Mindfulness.*

The possible similarity of the medians scores of depression obtained by men and women at the first passing time regardless of practice group was tested using the non-parametric Mann–Whitney U test. The result showed a significant difference in intersex medians (*U* = 2911, *p* = 0.004), with women having significantly higher depression scores than men. Another test was conducted to assess whether there was an initial difference in depression scores between the two practice groups. Thus, no significant difference was detected (*U* = 3227, *p* = 0.364).

The effect of time, group, and gender on the evolution of the resilience score over time was tested using a repeated measures ANOVA with an intra-subject factor. Results using a Greenhouse-Geisser correction show that time [*F*(1,83) = 12.691, *p* < 0.001] and group [*F*(1,83) = 4.322, *p* = 0.041] had an effect on the change in the resilience score. *Post hoc* test of Tukey revealed that this observation was only true for the mindfulness group [*t*(83) = −3.108, *p* = 0.013]. Gender had no effect on the evolution of resilience scores over time [*F*(1,83) = 2.36, *p* = 0.129]. The Wilcoxon signed-rank test showed that being physically active had a statistically significant change in depression score over time (Z = −1.983, *p* = 0.047) while mindfulness practice had no statistically significant change on the depression score over time (Z = −1.274, *p* = 0.203). Gender also had a significant effect on the change in depression score over time. Both men (Z = −4.006, *p* < 0.001) and women (Z = −6.591, *p* < 0.001) saw their scores decrease over time.

Resilience was negatively correlated with depression at the first (*r* = −0.491) and second (*r* = −0.448) handover times. Two linear regressions showed that in the first pass time, the resilience score explained 24.1% (*p* < 0.001) of the depression score. At the second stage, the resilience score explained only 20% (*p* < 0.001) of the depression score.

## Discussion

One of the primary objectives of this study was to investigate the evolution of the resilience and depression score according to the type of practice (e.g., physical activity or mindfulness) in which participants were engaged in during the first wave of the COVID-19 pandemic. Regarding the scores obtained on the CD-RISC, the results of the physical activity group showed that their score at the first and second measurement time remained stable over time. Conversely, although the mindfulness group had a lower initial score than the physical activity group, the mindfulness group’s score improved on the second measurement and equaled the score obtained by the physical activity group. Resilience is a practice-modifiable construct (Southwick et al., 2011) and the protective effects of physical activity and mindfulness can be enhanced with increased investment in practice (Carmody and Baer, 2008; Deuster and Silverman, 2013). Conversely, sedentary lifestyles are associated with greater vulnerability to a variety of chronic physical and mental health conditions (De Mello et al., 2013; Deuster and Silverman, 2013). It is possible that physical activity practitioners were proportionally more active in their practice in the pre-pandemic period than those engaged in mindfulness, which may explain the initial difference in resilience scores between the two groups. In addition, descriptive statistics from this study show a clear age difference between the two groups (*M*_*age–AP*_ = 25.1; *M*_*age–Mind*_ = 35.16), suggesting that engagement in physical activity occurs earlier than engagement in mindfulness, allowing more time for physical activity practitioners to develop resilience. It has also been shown that those engaged in mindfulness tended to be white women, aged 45–64, university graduates, with higher incomes, and more likely to be physically active (Burke et al., 2017). During the pandemic, many prevention messages were shared regarding the mental health of the population, which could hypothetically lead mindfulness practitioners to increase the time invested in their practice and thus allowing the improvement of their F-CDRISC resilience scores in the second testing. Since practice time was not examined in this study, more research needs to be done to validate this hypothesis.

The pandemic can be seen as a potentially traumatic event that can disrupt the human biopsychospiritual homeostatic balance point as defined by Connor and Davidson (2003). When individuals had to cope with the internal and external stressors generated by this extraordinary situation, they had to go through a process of reintegration that may result in a gain, stabilization, loss, or dysfunctional state of homeostasis. Participants who were physically active during the first wave recorded a score statistically equal to both the first and second test time. They recovered their initial state of biopsychospiritual homeostasis after having invested an effort to overcome the disturbance. In other words, their initial level of resilience allowed them to cope with the adversity encountered during the first wave of COVID-19 pandemic without being affected over the long term. Meditation is often used to regulate emotions or promote mental health, also in the case of disorders (e.g., depression, anxiety, stress) (Cramer et al., 2016; Pepping et al., 2016; Upchurch and Johnson, 2019). According to Connor and Davidson’s theory, the disruption in the state of biopsychospiritual homeostasis engendered by COVID-19 pandemic seems to have allowed mindfulness practitioners to grow and improve their resilience. As a result, those who engaged in mindfulness meditation were able to achieve a new state of biopsychospiritual homeostasis that was higher than initially. Research in the field of mindfulness has shown that it can act beneficially on at least three categories of different but potentially interdependent dimensions. These categories are affects, interpersonal dimensions, and intrapersonal dimensions (Davis and Hayes, 2011). Thus, among other things, mindfulness practice would make it possible to better manage emotional challenges, stress and relational conflicts as well as to have greater tolerance for negative affects (Carmody and Baer, 2008; Eubanks Gambrel and Keeling, 2010; Farb et al., 2010; Davis and Hayes, 2011). During the first wave of COVID-19 pandemic certain protective factors, such as optimism, support and social connectedness, were specifically highlighted to help people cope with the negative effects of the pandemic on physical and mental health (Chen and Bonanno, 2020). Mindfulness practitioners have been shown to be more optimistic than non-mindfulness practitioners. Furthermore, the practice of mindfulness would have a significant impact on the tendency to judge information more positively than negatively (Kiken and Shook, 2011).

The depression score measured twice during the first wave of the pandemic evolved in only one practice group. Indeed, statistical analyses showed that among physically active people, the depression score decreased between the two measurements. This result was not observed in mindfulness practitioners, who maintained a stable depression score over time. Exposure to numerous stressors such as isolation, social distancing or insecurity can have adverse effects on the well-being, emotional reactions that translate into distress or the development of psychiatric disorders (Brooks et al., 2020; Cullen et al., 2020; Pfefferbaum and North, 2020; Rubin and Wessely, 2020). Physical activity as well as mindfulness has been shown to have mental health benefits. Indeed, there is a positive correlation between physical activity level and mental health indicating that being physically active would improve mental health (Paluska and Schwenk, 2000). In this sense, De Mello et al. (2013) showed that a sedentary lifestyle can more easily lead to the development of symptoms of depression and anxiety. Conversely, investment in physical activity would have a protective effect on mental health and, more specifically, on the development of depression (De Mello et al., 2013). Physical activity and exercise would also have an antidepressant effect comparable to that of pharmaceutical treatments (Dinas et al., 2011). Some results suggest that the prevalence of depressive symptoms may have tripled between the pre-COVID-19 period and the pandemic (Ettman et al., 2020). Depression impacts, among others, the central nervous system and the immune system. Investing in physical activity can counterbalance the negative effects of depression by improving the anti-inflammatory process, the immune system, neurogenesis, and neuroprotection and by increasing endorphin secretion (Woods et al., 2020). Physical activity thus appears to have the potential to act at different levels, both in the prevention and treatment of depression. On the other hand, mindfulness allows the acquisition or reinforcement of certain protective factors acting on psychological functions during situations of intense stress. Thus, practicing mindfulness can have a positive effect on cognitive resilience, mental health, emotional balance and on reducing anxiety, depression and burnout risks (Conversano et al., 2020). However, in this study, an improvement in the depression score could not be observed, which may suggest that the practice of mindfulness only served to protect practitioners from an increase in this score. Although the resilience score increased over time in the mindfulness group, this did not result in an improvement in the depression score. In light of this result, further analysis was conducted which found that the resilience score explained only 20–25% of the variance of the depression score, which means that other variables may explain the relation between resilience score and depression score.

The second objective of this study was to assess the impact of gender on the depression and resilience scores recorded by the two groups. The results of this study show that women and men had significantly different initial levels of resilience, with women scoring lower on average. Conversely, they scored initially significantly higher on the BDI-FS-Fr and this, independently of the group to which they belonged. Gender had no effect on the resilience score, however, both men and women saw their depression scores decrease significantly over time. Here again, the resilience score explained just partially the depression score which could explain the difference measured in the evolution of those two variables. The hypothesis that there may be a difference between men and women concerning those two variables has already been tested in several studies. Bonanno et al. (2007) have argued that gender is a robust predictor of the level of resilience. In their study, women were more likely to have lower levels of resilience than men. Erdogan et al. (2015) also investigated the impact of gender on students’ resilience levels and came to the similar conclusion that men had significantly higher levels of resilience than their female peers. Female are at greater risk of experiencing psychological distress while men show better adjustment to stressful situations (Conversano et al., 2020). According to a WHO report, the higher prevalence of depression among women may be related to socially determined gender norms, roles and responsibilities (World Health Organization (WHO), 2002). Isolation, the closure of many educational, work, and leisure facilities, and travel bans impacted the economy during the COVID-19 pandemic (Nicola et al., 2020). It is likely that women have been more affected by these restrictions, which, by social norms, have more weight on their shoulders when it comes to managing the organization of the home or caring for children. Women may also be more at risk when it comes to layoffs. These different assumptions may be the source of many concerns that can cause more psychological distress for women than for men. The decrease in depression scores over time in women and men regardless of practice may be due to habituation to the situation or to the gradual return to normal that seemed to be on the horizon. The second handover was indeed carried out shortly after the Federal Council announced the reopening in several stages of certain businesses and cultural venues.

This study has some limitations. The sudden arrival of the coronavirus in Switzerland as well as the uncertainty about the evolution of the situation required that data collection for this research be done very quickly. Thus, the samples from both groups suffered from a high loss of subjects while the sample of mindfulness participants was already small at the beginning of the study. In the case of physical activity, the sample included mainly university students. It has already been shown that student populations were not always representative of the general population (Sears, 1986). The generalization of the results observed in this study must therefore be made with caution. A second limitation concerns subjective measures of depression and resilience scores. Indeed, CD-RISC and the BDI-FS-Fr are self-report questionnaires and can therefore lead to bias. Fisher and Katz (2000) have in fact argued that self-reported values can be influenced by social desirability bias and that this influence is positively correlated with the relative importance given to the object by the culture of the individuals responding to the questionnaire. In addition, several demographic data such as socioeconomic status, religion, ethnicity, marital status, education level, and medical and psychological status were not collected and could have impacted the measures of resilience and depression while providing more insight into the results obtained. Finally, this study suffers from a lack of information regarding the physical activity and mindfulness habits of participants. Indeed, more details on the actual practice of these two activities, such as the duration invested per day, the frequency and the content of the sessions would have allowed to better define the scope of the impact of these practices on mental and physical health. In future research, it would be relevant to investigate the processes by which Mindfulness, physical activity, and gender affect depression and resilience scores. In the case of Mindfulness, for example, it is impossible to know which protective factors were enhanced to achieve a higher resilience score. Social desirability bias could be mitigated by incorporating objective measures of resilience and depression, such as interviews, in addition to self-report questionnaires. Finally, future research could include a control group practicing neither physical activity nor mindfulness in order to better identify the causal relationship between the practice of these two activities and the results obtained.

## Conclusion and Practical Implications

This study is one of the first attempt, to our knowledge, to look at the effects of physical activity and mindfulness on resilience and depression during the COVID-19 pandemic. The unique health context in which this study was conducted provides an opportunity to expand knowledge of the effects of physical activity and mindfulness on depression and resilience. Thus, during a potentially traumatic event, the practice of mindfulness would make it possible to increase one’s level of resilience, which plays a preponderant role in coping with adversity. Conversely, being physically active would allow a high level of resilience that remain stable over time and a decrease in the depression score. The difference in resilience and depression scores between men and women has already been observed in other studies (Bonanno et al., 2007; Erdogan et al., 2015; Conversano et al., 2020) and may be due to social norms that impose more family responsibilities on women and to women’s greater vulnerability to economic instability and thus unemployment. The results observed can help direct prevention and health promotion organizations to people at higher risk of developing depression during a health crisis. Both mindfulness and physical activity seem to provide protective effects to cope with adversity, they also have already demonstrated numerous beneficial effects on physical and mental health in everyday life (Bränström et al., 2010; De Mello et al., 2013). It may be relevant to promote the practice of mindfulness among younger people in order to encourage the development of the protective factors it can provide. From a practical point of view, the results observed in this research suggest that engaging in physical activity or mindfulness would allow one to be resilient to potentially traumatic situations such as a pandemic. Thus, it may be relevant to promote health programs that include mindfulness and physical activity to cope with the potentially traumatic effects of a pandemic. The protective factors developed or improved through the practice of these two types of activities could also be transposed to situations of adversity encountered in everyday life.

## Nomenclature

### Resource Identification Initiative

**BDI:** Beck’s Depression Inventory
**BDI-FS-Fr:** Beck’s Depression Inventory Fast-Screen France
**f-CD-RISC:** French version of the Connor and Davidson Resilience Scale
**Mind:** Mindfulness
**PA:** Physical Activity
**PTE:** Potentially Traumatic Event
**PTSD:** Post-Traumatic Stress Disorder
**RISC:** Connor and Davidson Resilience Scale
**WHO:** World Health Organization

## Data Availability Statement

The raw data supporting the conclusions of this article will be made available by the authors, without undue reservation.

## Ethics Statement

The study was conducted in accordance with the Declaration of Helsinki and the Code of Ethics and Conduct of the British Psychological Society. All participants were informed about the anonymization of the data, and about each procedural step of the research, from data collection to analysis and publication. Ethical approval for the recruitment of questionnaire respondents was granted by the Research Ethics Committee of the University of Graz. The patients/participants provided their written informed consent to participate in this study.

## Author Contributions

RA and MB conceived and designed the study. LS collected and organized the database and performed the analysis. RA, LS, and MB co-wrote the manuscript and contributed to manuscript revision. All authors read and approved the final submitted version.

## Conflict of Interest

The authors declare that the research was conducted in the absence of any commercial or financial relationships that could be construed as a potential conflict of interest.

## Publisher’s Note

All claims expressed in this article are solely those of the authors and do not necessarily represent those of their affiliated organizations, or those of the publisher, the editors and the reviewers. Any product that may be evaluated in this article, or claim that may be made by its manufacturer, is not guaranteed or endorsed by the publisher.
